# FynT tyrosine kinase as a potential modulator of microglial activation and inflammasome signaling in neurodegenerative diseases

**DOI:** 10.1002/alz70855_106741

**Published:** 2025-12-24

**Authors:** Michelle GK Tan, Frances TW Lim, Clara YB Low, Helen L Ong, Mitchell KP Lai

**Affiliations:** ^1^ Singapore General Hospital, Singapore, Singapore; ^2^ National University of Singapore, Singapore, Singapore; ^3^ Wolfson Centre for Age‐Related Diseases, King's College London, London, United Kingdom; ^4^ Memory Aging and Cognition Center, National University Health System, Singapore, Singapore

## Abstract

**Background:**

We reported that FynT tyrosine kinase was significantly upregulated in postmortem brains of patients with Alzheimer's disease (AD) and Lewy body dementia (LBD), as well as in aged P301S tauopathy mice. Its expression positively correlated with tau pathology and neuroinflammation. Given that Fyn kinase has been implicated in priming and activating the microglial NLRP3 inflammasome, thereby amplifying neuroinflammation, and that FynT was predominantly expressed in microglia, we hypothesize that FynT may modulate microglial activation and inflammasome signaling, exacerbating neuroinflammation and neurodegeneration.

**Method:**

Using real‐time RT‐PCR, we analyzed the expression of FynT, microglial and inflammasome signaling markers in postmortem brain tissues from AD, LBD and control cases. Additionally, we examined aged males P301S tauopathy mice, with and without FynT depletion, to assess changes in microglial and inflammasome‐related gene expression.

**Result:**

Microglia, activation and inflammasome signaling markers were significantly upregulated in AD and LBD brains compared to controls and positively correlated with FynT expression. In P301S mice, FynT depletion led to a significant reduction in microglial activation and inflammasome‐related markers, suggesting a direct role for FynT in neuroinflammatory processes.

**Conclusion:**

Our findings indicate that FynT tyrosine kinase contributes to microglial and inflammasome activation, promoting neuroinflammation and neurodegeneration. Targeting FynT depletion may represent a promising therapeutic strategy to mitigate neuroinflammatory processes and slow the progression of AD and related neurodegenerative diseases.

